# Low-cost equipment for the evaluation of reach and grasp in post-stroke individuals: a pilot study

**DOI:** 10.1186/s12938-020-0758-7

**Published:** 2020-03-04

**Authors:** Camila L. A. Gomes, Roberta O. Cacho, Viviane T. B. Nobrega, Ellen Marjorie de A. Confessor, Eyshila Emanuelle M. de Farias, José Leôncio F. Neto, Denise S. de Araújo, Ana Loyse de S. Medeiros, Rodrigo L. Barreto, Enio W. A. Cacho

**Affiliations:** 1 School of Health Sciences, Federal University of Rio Grande do Norte (UFRN), Santa Cruz, RN Brazil; 2 Federal Institute of Rio Grande do Norte (IFRN), Santa Cruz, Brazil; 3 Research Department, Federal Institute of Rio Grande do Norte Campus, Santa Cruz, RN Brazil

**Keywords:** Stroke, Physical therapy, Upper extremity, Hand strength, Equipment failure analysis

## Abstract

**Background:**

Reach–grasp movements are motor components commonly affected after stroke and directly related to the independence of these individuals. Evaluations of these activities can be performed using clinical instruments and assessed by detailed and costly kinematic analyses. The aim of this study was to develop an analysis of reach–grasp movements in post-stroke patients using a simple, inexpensive, and manageable instrument.

**Results:**

A Mann–Whitney test was used to compare paretic and non-paretic limb motor performance. A statistically significant difference was found between the variables of total time (*p* = 0.02) and speed to reach target 3 (*p* = 0.04) for task 1, while in task 2 significance was found only in the aspect of speed to reach target 2 (*p* = 0.04). The correlation between clinical tests and variables of tasks was then performed using Spearman’s rank correlation coefficient. At task 1, when compared with the REACH instrument, the close target sub-item; there was a high positive correlation between the parameters of total time (*p* = 0.028), target velocity 3 (*p* = 0.028), and target acceleration 3 (*p* = 0.028). Another instrument that showed a high positive correlation with the target time 3 (*p* = 0.01) and target acceleration 3 (*p* = 0.028) variables was the Box and Block Test. When correlated, the data between the task 2 variables and clinical instruments did not present statistically significant data.

**Conclusion:**

Our instrument—the Temporal Data Acquisition Instrument—TDAI—fulfilled the expected objectives and can be used as an option to evaluate the movements of reach and grasp of upper limb post-stroke, using an easy and fast application, without the need for calibration.

*Trial registration* Trial Registration: Research Ethics Committee of the Trairi School of Health Sciences—Number 2.625.609, approved on April 13, 2018; Brazilian Registry of Clinical Trials—RBR-4995cr approved on July 4, 2019 retrospectively registered (http://www.ensaiosclinicos.gov.br/rg/RBR-4995cr/)

## Background

The functional independence of individuals after stroke is directly influenced by the ability to perform reaching and grasping movements successfully [1]. However, the performance of these motor activities is commonly affected after stroke, leading to slower and segmented movements, and these, in turn, may be associated with compensatory movements of structures such as the shoulder and trunk [2].

Reach and grasp movements involve the coordination of the fingers and thumb, previously positioned according to the size, shape, and function of the object that are combined with the movement of the arm toward the object and the control of the force to hold the target and keep it in the hand during its transport [1, 3].

Several conventional and standardized clinical measures aim to evaluate reach and grasp activities in post-stroke subjects [4]. Instruments, such as the Fugl Meyer Functional Performance Scale (FM), Box and Blocks Test (BBT), and Action Research Arm Test (ARAT), aim to quantify movement changes through observational analyses, further classified on either ordinal or nominal scales [2, 5].

The assessment of reach and grasp through clinical tests, when applied by trained professionals, allows the verification of psychometric properties and their effects on daily activities. In addition, the evaluation uses instruments that are inexpensive, simple, and quick to apply [6]. However, the results of these instruments have a gap for control and measurement of procedures to provide more meaningful and detailed results, not only for distinguishing different patterns of impairment and compensation strategies but also for analyzing follow-up during treatment of these motor activities [2, 7].

Kinematic laboratory analyses allow the objective and precise examination of the points that need to be addressed to improve reach and grasp movements [7, 8]. These instruments analyze temporal variables that are not observed directly by clinical instruments. However, the kinematic assessment available and consolidated products are costly [9, 10].

For this reason, because of the scarcity of free or low-cost equipment, it is necessary to improve studies involving the construction of devices that allow evaluations of different parameters of reach and grasp movements using conventional clinical instruments for clinical and scientific purposes.

Therefore, the aim of this study was to develop an inexpensive, manageable tool for assessing reach and grasp movements that enables the analysis of aspects not addressed by conventional clinical measures and provides an alternative to expensive kinematic analysis equipment.

## Results

The data related to the sample characterization are shown in Table 1.

**Table 1:** Demographics and clinical characteristics of participants (*n* = 8)

Characteristic	Value	
	*n* (%)	Median (1Q/3Q)
Sex, *n* (%)		
Female	3 (37.5)	
Male	5 (62.5)	
Age, in years		66 (59/68.75)
Time onset stroke, in months		44 (11.25/93.00)
Affected hand, *n* (%)		
Right	5 (62.5)	
Left	3 (37.5)	
FMA-UE		50 (44.75/54.25)
Nottingham Sensorial test		150.5 (138.5/153.25)
MMSE		25 (21/28.25)
REACH close target		17.5 (11.75/18)
REACH distant target		16 (11.75/17.25)
BBT		33 (21.25/37.5)
ARAT		55 (48.75/56.25)

All subjects were ischemic stroke

All subjects were right-handed

*n* number, *1Q* first quarter, *3Q* third quarter, *FMA*-*UE* Fugl Meyer Assessment for the Upper Extremity, *MMSE* Mini-Mental Status Examination

In Table 2, the kinematic variables obtained by the TDAI system showed better performance in the time and in the velocity aspects when comparing the results between the most affected limb and the least affected limb, related to task 1.

**Table 2:** Comparison of motor performance with paretic and non-paretic limb in the accomplishment of task 1

Variable	Mann–Whitney test	
	Paretic limb Median (1Q/3Q)	Non-paretic limb Median (1Q/3Q)
Time	3.34 (2.94/3.78)	2.77 (2.58/2.91)*
Time target 1	1.36 (1.12/1.50)	1.18 (0.94/1.25)
Time target 2	2.27 (2.05/2.57)	2.00 (1.65/2.13)
Time target 3	3.37 (2.98/3.80)	2.80 (2.58/2.91)*
Velocity 1	34.34 (29.43/43.04)	40.12 (39.51/48.39)
Velocity 2	18.57 (14.31/22.18)	21.87 (18.94/23.34)
Velocity 3	7.08 (5.94/9.71)	11.09 (9.52/11.75)*
Acceleration 1	49.40 (45.18/85.66)	78.40 (70.78/105.87)
Acceleration 2	58.75 (30.28/75.42)	68.77 (53.12/85.63)
Acceleration 3	15.83 (11.53/40.93)	32.78 (25.11/39.13)
Effectiveness	100%	100%

*1Q* first quarter, *3Q* third quarter

*p values: level of significance: ≤ 0.05

While in task 2 (Table 3), the velocity variable obtained significant difference between the upper limbs.

**Table 3:** Comparison of motor performance with paretic and non-paretic limb in the accomplishment of task 2

Variable	Mann–Whitney test	
	Paretic limb Median (1Q/3Q)	Non-paretic limb Median (1Q/3Q)
Time	2.54 (2.26/3.87)	2.36 (2.25/2.82)
Time target 1	1.52 (1.23/2.36)	1.44 (1.29/1.65)
Time target 2	2.54 (2.26/3.87)	2.36 (2.22/2.82)
Velocity 1	30.07 (21.47/37.24)	35.55 (30.71/39.58)
Velocity 2	12.91 (10.29/15.19)	19.29 (14.92/23.38)*
Acceleration 1	40.49 (23.55/65.99)	55.33 (42.66/69.00)
Acceleration 2	28.75 (20.25/35.49)	44.80 (35.26/66.49)
Effectiveness	100%	100%

*1Q* first quarter, *3Q* third quarter

*p values: level of significance: ≤ 0.05

The kinematic variables in tasks 1 showed some correlation with the clinical instruments used, as shown in Table 4. However, regarding the second task, none of the variables correlated with conventional clinical instruments.

**Table 4:** Correlation between variables of Task 1 and conventional clinical instruments

Variable	Spearman’s rank correlation coefficient			
	REACH close target	REACH distant target	BBT	ARAT
Time	0.76*	0.55	0.69	0.25
Time target 1	0.43	0.14	0.64	0.25
Time target 2	0.21	0.06	0.54	0.36
Time target 3	0.67	0.33	0.83*	0.18
Velocity 1	0.48	0.21	0.69	0.18
Velocity 2	0.12	0.21	0.14	0.10
Velocity 3	0.76*	0.49	0.59	0.36
Acceleration 1	0.48	0.21	0.69	0.18
Acceleration 2	0.07	0.07	0.28	0.50
Acceleration 3	0.76*	0.45	0.71*	0.40

*BBT* Box and Blocks Test, *ARAT* Action Research Arm Test

*p values: level of significance: ≤ 0.05

## Discussion

This study developed an assessment methodology for reaching, grasping, and pointing movements, which enables the analysis of parameters that are not observed by clinical instruments and offers an alternative to high-cost kinematic analysis equipment [7–10].

Reach–grasp assessments in post-stroke individuals are performed by clinical instruments or complex kinematic motion capture systems [5, 11]. Conventional clinical evaluations usually use daily activities, as well as being practical and for standard application. However, they have superficial and limited results, as they are little subject to the subtle alterations and may not be sensitive enough to identify the residual deficits in these patients [6, 7, 11–16].

Kinematic assessment instruments, in turn, promote more detailed, objective, and accurate measurement [2, 6, 17]. However, they are expensive, bulky pieces of equipment that require time to adjust and calibrate, making them less practical for use in clinical or laboratory daily life [7, 9].

This study proposed to develop a system that had simple handling, easy displacement, fast application in the clinical environment, and low cost. The TDAI achieved this goal. Out equipment can be quickly assembled and disassembled in less than 5 min, and weighs about 300 g, making it easy to transport and was built with less than $25. No time is required for calibration, and UE assessment can be applied in 20–30 min (including time for adjustment and participant instructions). In addition, it has a low financial investment for preparation and maintenance. The equipment enables the collection of essential data parameters needed for the evaluation of motor performance, such as average speed, average acceleration, duration time, and movement effectiveness [8, 16, 17].

The analysis of temporal variables is usually restricted to the laboratory environment. They cannot be observed by conventional clinical instruments, but can only be verified by kinematic analysis equipment. These occur because they require controlled and customized environments, and because they have complex and expensive use, for this reason not being used in the clinical environment [10, 18].

Thus, these temporal measures are important at the clinical environment since they can be used in the therapeutic follow-up and the study of new therapeutic approaches, since these kinematic variables are not observed directly by clinical instruments and, usually, they are altered after stroke. The coordination of reach and grasp movements is complex [11] and these individuals have difficulties in planning and controlling specific aspects of their movements, such as the speed and acceleration, which hinders harmonious motor performance and relearning movements [19–21].

Adjusting speed control during a motor action is essential for performing quality motor skills movements [17]. This variable, when evaluated, allows the verification of the movement performance during repetitions and reveals aspects of the performance improvement [16]. Studies indicate that decreasing speed indicates worsening motor performance [1, 22–24].

Acceleration presents few reports of changes after post-stroke therapeutic interventions despite being an important aspect analyzed in kinematic evaluations [21, 25, 26]. This measure reflects the smoothness of motor activity and implies faster movement onset and greater motor control throughout the action [11, 27–29].

Regarding the duration of movement, this parameter is considered an aspect of temporal efficiency [16]. It can be defined as the time to perform a particular motor activity, starting from the initial stimulus interval until the completion of the task [30]. Moreover, this measure also reveals data related to the motor performance of the evaluated individual where a decrease in the time to complete the activity indicates an improvement in the movement performance [16].

The equipment enables the calculation of not only the total time to complete the task but also the time to reach each of the targets. This feature allows the observation of the variation of the interval between the targets. Since this variation is another indicator of execution time, it reveals which movements demand shorter or longer times to be performed by the participant.

Another variable that can be observed by the equipment is effectiveness, a measure that indicates the individual’s ability to produce the motor result successfully. The evaluation of this parameter allows following the performance evolution along with the repetitions, besides allowing observation of the necessary adaptation or evolution of the chosen motor task [31].

In this study, the sample obtained 100% effectiveness on both tasks (T1 and T2). This response is believed to have occurred because of the inclusion criteria adopted (to be able to perform the flexor and extensor synergies according to the FMA-UE), which selected patients with mild and moderate impairment.

The evaluation of these temporal parameters allows to visualize subtle differences, as can be observed in this study. All individuals evaluated were in the chronic phase of the disease and had high functioning, approaching the maximum score of conventional clinical instruments. These results may be overlooked or difficult to discover due to the ceiling effects of functional assessment tools [15]. However, from the TDAI evaluation, it was possible to compare the performance of the paretic limb with the non-paretic limb and to verify that there was still a capacity to be explored in all individuals who participated in this study, enabling a comprehensive and detailed assessment of motor changes in the UE even with those with mild impairment.

In addition, within the stroke group, people with moderate impairment had more marked kinematic deviation controls compared to people with mild impairment [11]. Therefore, individuals with higher levels of motor impairment would present more statistically significant variables when comparing the motor performance of the paretic limb with the non-paretic limb.

We believe that the TDAI can be used not only in comparative aspect (pre-/post-treatment) of reach and grasp, but also in the training of these movements. In addition, it can be also used as a parameter for the adaptation or progression of the exercises, since our equipment analyzes not only efficiency, but also effectiveness variables. It is still possible to increase the capacity of the TDAI analysis using it in conjunction with other devices, such as surface electromyography (sEMG) and/or accelerometer, besides generating the equipment parameters, co-contraction measures (agonist and antagonist muscles), reaction times, and peak speeds, for example.

In this study, we also related the results of TDAI with the conventional clinical tests, but few correlations were observed. We credit this to the fact that the evaluated aspects of human movement are different from those measured, since conventional clinical instruments have results on ordinal scales (REACH and ARAT) or number of cubes transported (BBT), while the device has numerical results corresponding to temporal variables [27, 32, 33].

As these assessments observe different aspects, thus bringing different analyzes of the individual’s condition and motor capacity, we suggest the use of TDAI as a complement to conventional clinical instruments, promoting a standardized and more detailed and less subjective results regarding the motor performance evaluation of reach and grasp movements post-stroke.

## Limitations and future research

Considering the use of the equipment compared with the instruments of kinematic analysis, the present study does not provide an analysis of all aspects addressed in kinematic programs and is therefore not as detailed. However, the device can be used in conjunction with other equipment, such as an EMG, to obtain other motor performance variables. Further research using equipment such as a reach and hold training strategy is suggested, as well as its application together with an EMG for UE motor performance assessment.

## Conclusion

In this study, we defined a set of parameters for the TDAI assessment of UE motor performance after stroke. The equipment elaborated in this study captures and processes the temporal variables of the motor performance, which are not observed by the conventional clinical instruments and reveals details that allow to identify even minor differences, being able to use the UE as a reference. In addition, due to its low weight and easy assembly and adjustments, beyond that to a short application time, it allows temporal assessments to be carried out also in the clinical environment, where commonly just conventional clinical instruments are used, allowing more detailed analyzes not only with purposes for research as well as in the daily life of rehabilitation.

The TDAI fulfilled the expected objectives; can, therefore, be used as an option for the low assessment of post-stroke UE reach and grasp movements, easy and quick to apply, without the need for calibration; and is portable so the evaluator can have access to it at all times. Thus, monitoring these temporal variables in the clinical setting through TDAI enables the therapist to be able to plan, adjust, and progress a personalized treatment plan.

## Methods

The Temporal Data Acquisition Instrument (TDAI) system was designed by the authors and provide an automated interface with data acquisition, capture, and processing data of temporal human movement variables (movement times, mean velocity, mean acceleration, and movement effectiveness) from the upper limb extremity (UE) using two motor skills (reach–point: T1 and reach–grasp–fit: T2). The TDAI system was built to be used easily by the therapist in a clinical setting, has a low cost of installation and maintenance (was designed and built with investment less than $25), and is capable of producing temporal kinematic information on reaching, grasping, and pointing movements.

### Equipment design

The TDAI allows the assessment of reach–grasp and pointing movements by two tasks. In T1 (reach-point), the participant must reach and touch three targets arranged in an “L” shape. While in T2 (reach–grasp–fit), the individual must carry a glass between two distinct targets spaced 15 cm apart.

The material is composed of a single board, rectangular (28.19 cm in length, 20.35 cm in width, and 5 mm in depth). At the anterior part, the targets that must be reached in each activity are indicated: three targets arranged in an “L” shape (T1) and two distinct circumferences of 5 cm in diameter (T2).

**Fig. 1:**
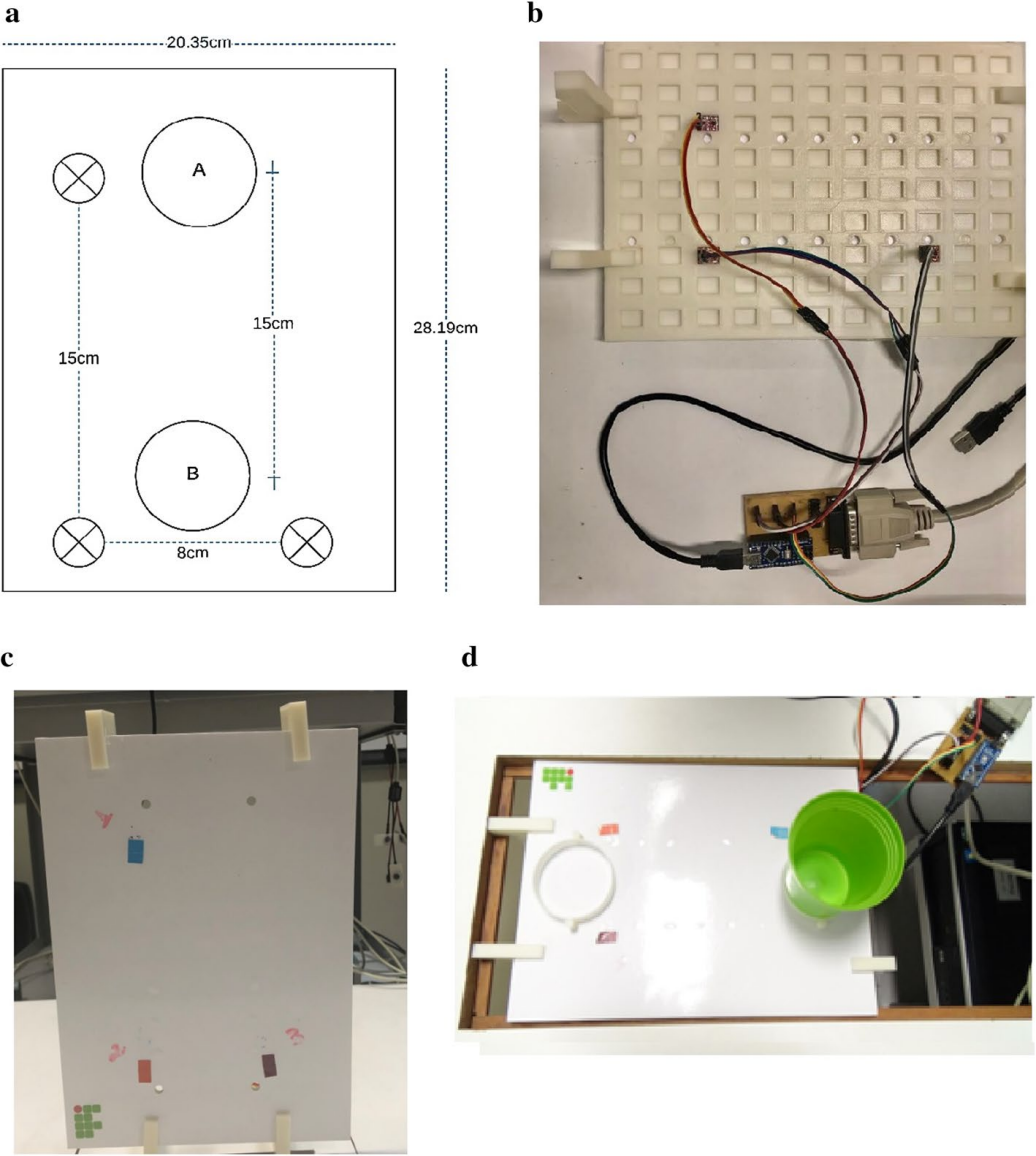
Front and back of the board. TDAI consists of a single plate, that can be positioned vertically (T1) or horizontally (T2). On the front of the board (**a**) the targets of each activity are indicated (targets represented by “x”—T1; items “A” and “B”—T2). The back of the board (**b**) is intended for fitting the capacitive sensors, referring to the selected targets on the front of the board. Targets related to task 1 were represented by numbers to indicate the sequence to be followed, as well as by rectangles of different colors (**c**). The targets of task 2 consisted of two circles, where the cup was to be transported from the farthest target and fitted to the nearest target (**d**)

The back part of the board is used for positioning capacitive sensors by snapping them into hollow parts (rectangles measuring 14.7 mm × 11 mm × 4 mm), in reference to targets positioned on the front of the plate (see Fig. 1). However, as the entire back part of the board is made up of hollow rectangles, it is possible that the capacitive sensors are positioned not only according to the tasks determined by the authors, but also to use the arrangement that is desired.

The TDAI has two structures that allow its positioning on the table according to the activity. It can remain vertically (T1) or be placed horizontally (T2). The main board and its accessories (cylinders and bracket) were constructed of poly-plastic (PL) filaments.

The equipment contains a circuit referring to capacitive sensors, components used for the acquisition of time data. They are inserted into the rectangular cavities, positioned according to the activity to be performed. The capacitive touch sensor (11 mm width × 14.5 mm length × 2.3 mm height) was used to replace the direct button switch to ensure user accessibility.

**Fig. 2:**
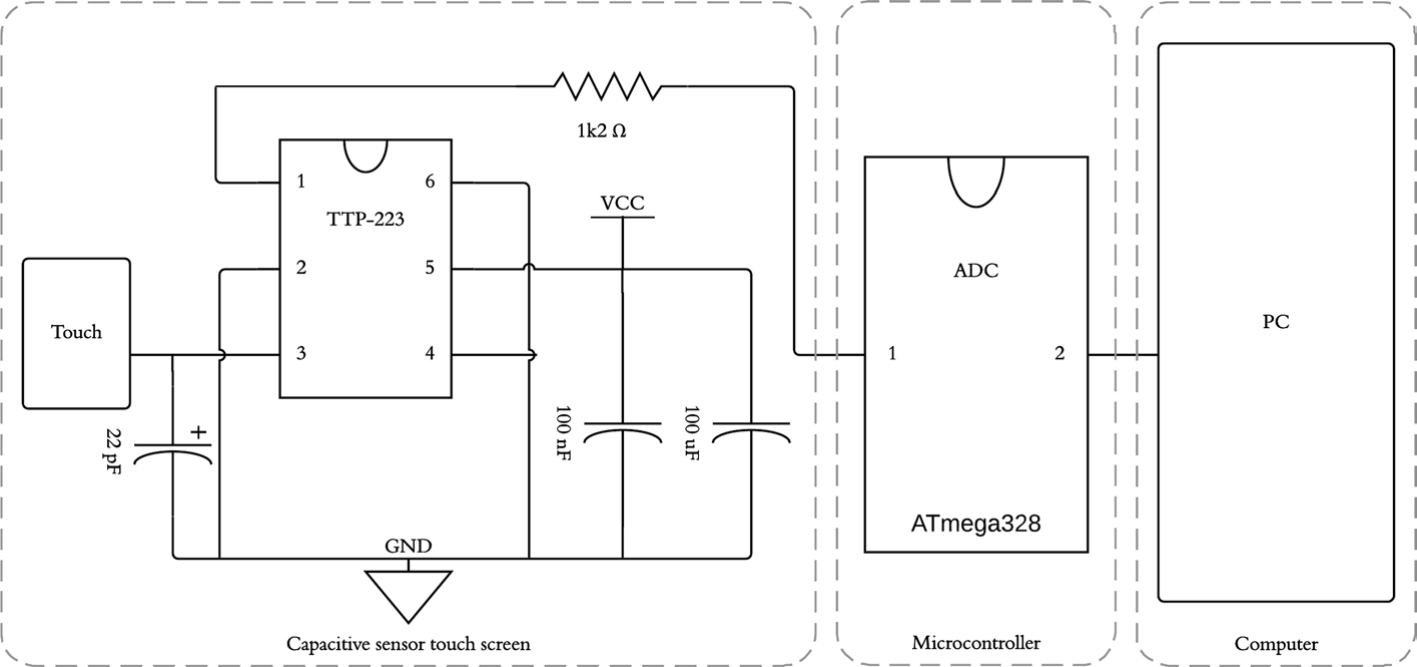
Signal conditioning circuit of the sensor. Sensor input voltages range from 2 to 5.5 V DC. The touch module TTP-223 processes the information with a 16-MHz clock, being sensitized ≤ 60 ms after contact. The 22-pF capacitor adjusts sensor sensitivity; 100-nF and 100-uF capacitors stabilize input voltages, eliminating high- and low-frequency noise. Communication with the computer is UART, using the I2C protocol

Sensor input voltages range from 2 to 5.5 V DC. The Touch TTP-223 module has a maximum response time of 60 ms after incitation. This component is responsible for identifying the individual’s touch and sending the electrical impulses from the incitement to the ATmega328 microcontroller that processes the information with a 16-MHz clock, which ensures system reliability (see Fig. 2).

### Evaluation by the TDAI system

**Fig. 3:**
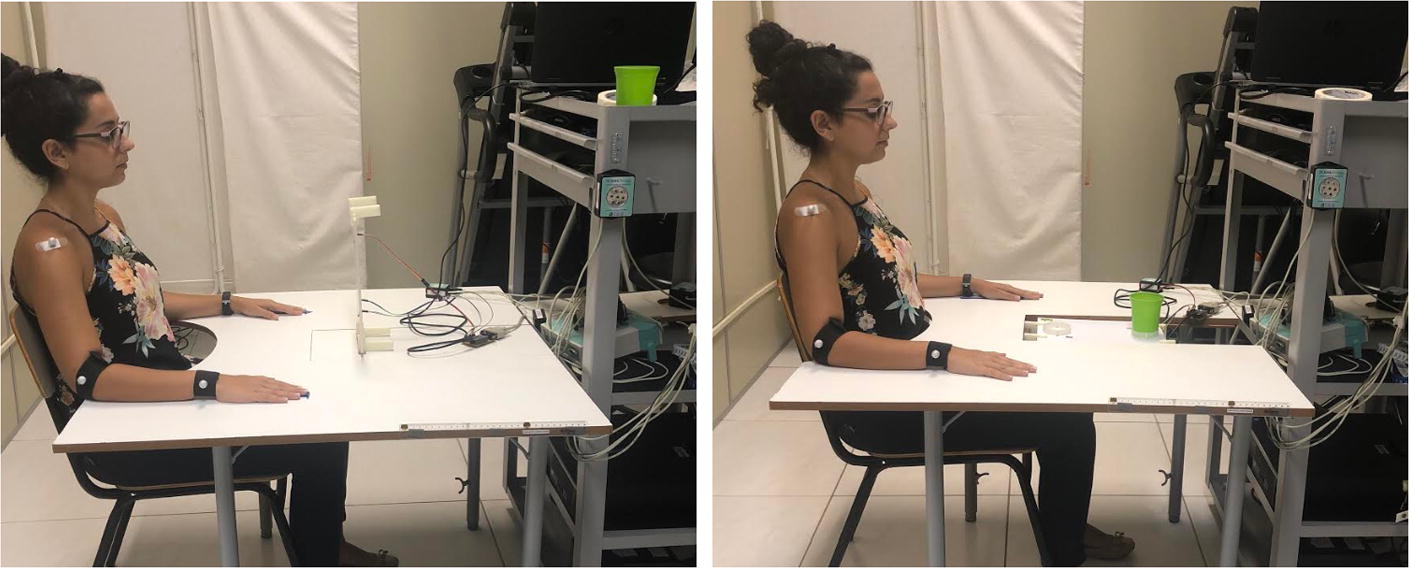
Participant positioning for TDAI assessment. To perform both tasks the individual is positioned in the same way, and the adjustments are made only on the platform, where for task 1 it is placed vertically (left image), while for task 2 the platform remains horizontal (right image)

The assessment using the TDAI consists of a single session protocol, lasting 20–30 min, where two reaching and grasping activities are performed with the affected UE. The clinical protocol was performed with the patient sitting, with the back-supported and trunk-free (without restrictions), facing a table with adjustable height, and the elbow positioned at 90° of flexion, the shoulder at 0° and the hand on the table at a demarcated point (Fig. 3).

Before the start of each task, simple verbal instructions and a demonstration were passed to the evaluated patients. Sixteen repetitions of each task were performed for each upper limb, with an interval of 15 s between each repetition and 3 min between each type of task. Each repetition was preceded by a sound trigger (from data acquisition plate), issued by the evaluator, as a reference to the participant to start the movement with his usual velocity.

For T1 (reach–point), the equipment is vertically fitted to the table, away from the margin equivalent to 90% of arm’s length—the distance measured between the axillary line and the styloid process of the radius from the sternum. Capacitive sensors are positioned so that the targets are in the shape of the letter “L,” where the distance between the first and second target is 15 cm (from its center) and 8 cm between the second and third targets.

For the accomplishment of T2 (reach–grasp–fit), the subject remains in the same position. However, modifications are made to the equipment: the capacitive sensors are repositioned so that they are straight and spaced at 15 cm. The main board is fitted to the table horizontally, and two cylinders (6 cm in diameter) are fitted into the front of the plate, in the same direction as the sensors, forming two different targets—where the farthest remains at 90% of arm’s length from the table edge. After the firing sound, the subject should reach for a glass (5 cm in diameter, 8 cm high), fitted to the farthest target, hold it, carry it, and fit it to the nearest target. At the end of each repetition, the patient returns to the starting point, and the instructor returns the cup to target 1.

### Clinical outcome measures

The TDAI processes information about the time to reach each target (s), total activity time (s), mean speed (cm/s), mean acceleration (cm/s^2^), and effectiveness (number of hits). These functions are performed on a practical, fast, economical, and manageable piece of equipment that does not require calibration or maintenance.

The variables are found using simple equations and by performing the routine to obtain the data in the spreadsheet software. The values for movement time are divided into the total time and times to reach targets 1, 2, and 3 (T1) and times to reach targets 1 and 2 (T2).

1 $$\Delta t = t - t_{o}$$

In Eq. 1, ∆*t* is the time value to be found, where *t*_o_ refers to the time of the beep and *t* the time the target has been reached. Therefore, this equation is used in three moments for T1 (target time 1, target 2, and target 3, where the latter also corresponds to the total time) and in two moments for T2 (target times 1 and 2, the latter also being equivalent to the total time).

Data related to the average velocity were obtained using Eq. 2.

2 $$V = \frac{\Delta d}{\Delta t}$$

where ∆*d* refers to the straight line distance from the table and target mark 1 (of the task in question), and ∆*t* corresponds to the time variation. This result is used to obtain the mean acceleration values, according to Eq. 3.

3 $$a = \frac{{\overline{\Delta V} }}{\Delta t}$$

In Eq. 3, ∆*V* refers to speed variation, while *t* refers to time variation. Efficacy values are obtained as a percentage, where 100% is 16 hits (all repetitions).

### Experimental protocol

The experimental protocol was carried out at Trairi Health Sciences Faculty (Facisa-UFRN), located in Santa Cruz-Rio Grande do Norte–Brazil. Eight patients with a clinical diagnosis of stroke, who were capable of performing flexor/extensor synergy movements related to the Fugl Meyer Assessment for Upper Limb Extremity (FMA-UE) with a score between 1 and 2, had an absence of sensory alteration in UE evaluated by the Nottingham Scale, or the presence of cognitive impairment assessed by the Mini-Mental Status Examination—MMSE (cutoff 20 points for illiterate; 25 to 28 for schooled [34]). All patients experienced a single unilateral stroke and should be over 18-year old.

A prior clinical evaluation (FMA-UE, Nottingham Scale, and MMSE) was performed to characterize the sample and inspect the inclusion and exclusion criteria and lasted 40 min. Subsequently, a trained therapist used specific clinical instruments (REACH Performance Scale, BBT, and ARAT) intended for the analysis of reach and grip movements (30 min for testing). The Reach Scale (REACH) focuses on compensatory strategies that are used during the transport phase in the range of motion and is defined by the beginning of the movement until the object is reached.

The test is divided into two sub-items, which are the near target (1 cm from the table’s edge) and distant target (30 cm from the table’s edge) [35]. Each subcategory evaluates six components: trunk dislocation, movement fluidity, shoulder movements, elbow movements, and grip. Each component ranges from 0 to 3 (where 0 indicates maximum compensation and 3 is equivalent to normal movement), corresponding to a maximum score of 18 points [35].

The BBT consists of a manual dexterity test where a wooden box is used, divided in half in two parts by a partition higher than the edges of the box. Evaluation occurs by the number of wooden cubes (2.5 cm) carried from side to side of the box for 1 min. The test is performed primarily with the unaffected limb, followed by the compromised limb [36].

Finally, the ARAT, also known as the Upper Limb Extremity Action Test, has 19 items that evaluate complex grip-related UE activities. The score ranges from 0 (no movement can be performed) to 57 (indicating normal motor performance). This scale does not allow classifying subjects as normal, mild, or severely limited [37].

### Statistical analysis

BioEstat version 5.3 was used for data analysis. The normality of the data was evaluated using the Shapiro–Wilk test, and non-parametric tests were applied. The study population and clinical characteristics were defined using descriptive statistics. The ability to discriminate changes resulting from stroke was verified with the Mann–Whitney test that was used to compare paretic and health limb motor performance. A Spearman’s rank correlation coefficient (for this, an average of 16 repetitions of each task was performed) was performed to observe the correlation between the measurements obtained from the task variables in the TDAI system with the clinical instruments. The following correlation classification was used; no or very low: *p* = 0–0.25; low: *p* = 0.26–0.40; moderate: *p* = 0.41–0.69; high: *p* = 0.70–0.89; very high: *p* = 0.90–1.0 [38].

## Data Availability

The authors declare that the availability of data and materials is not applicable, because this study focuses in the equipment development using variables of publicly available upper limb assessment.

## Abbreviations

FMA: Fugl Meyer Assessment
BBT: Box and Blocks Test
ARAT: Action Research Arm Test
T1: Task 1
T2: Task 2
UE: Upper Limb Extremity
MMSE: Mini-Mental Status Examination
TDAI: Temporal Data Acquisition Instrument
sEMG: Surface electromyography
UFRN: Federal University of Rio Grande do Norte
